# The We Can Quit2 Smoking Cessation Trial: Knowledge Exchange and Dissemination Following a Community-Based Participatory Research Approach

**DOI:** 10.3390/ijerph19042333

**Published:** 2022-02-18

**Authors:** Stefania Castello, Catherine Darker, Joanne Vance, Nadine Dougall, Linda Bauld, Catherine B. Hayes

**Affiliations:** 1 Public Health & Primary Care, Institute of Population Health, Trinity College Dublin, D24DH74 Dublin, Ireland; scastello@unc.edu.ar (S.C.); darkerc@tcd.ie (C.D.); 2 Community Programmes, Irish Cancer Society, D04VX65 Dublin, Ireland; joannevancecronin@icloud.com; 3 School of Health and Social Care, Edinburgh Napier University, Edinburgh EH11 4BN, UK; n.dougall@napier.ac.uk; 4 Usher Institute and SPECTRUM Consortium, College of Medicine and Veterinary Science, University of Edinburgh, Edinburgh EH16 4TJ, UK; linda.bauld@ed.ac.uk

**Keywords:** knowledge exchange and dissemination, smoking cessation, women, socioeconomic disadvantage, pilot randomised controlled trial (RCT), pilot cluster randomised trial (cRCT), community-based participatory research (CBPR)

## Abstract

Background: ‘We Can Quit2’ pilot randomised controlled trial determined the feasibility [of conducting a community-based trial of We Can Quit, a peer-delivered stop-smoking programme (group support, combination nicotine replacement therapy (NRT), and tailored individual support) for women living in socioeconomically disadvantaged areas in Ireland. Lessons from a knowledge exchange (KE) workshop that reengaged trial stakeholders are presented. Methods: The trial dissemination plan included invitation of community, regional and national stakeholders (*n* = 176) to a KE interactive workshop, who received an accessible brief beforehand. Trial findings were presented. Enhancements to community engagement, participants’ recruitment and retention, and policy priorities arising from the research were discussed. Field notes and responses to a post-event anonymous questionnaire were analysed using thematic content analysis. Results: Workshop attendees (41/176, 23%) recommended: dedicated additional time to engage community stakeholders; social prescribing pathways to enhance recruitment; more adaptation of trial-related information and assistance in completion of data forms for low literacy individuals; encouraging women to join healthy community programmes to facilitate retention and sustainability; removal of barriers to access NRT; and ongoing provision of cessation services tailored to disadvantaged groups. Conclusions: The findings are relevant to the implementation of other community-based health interventions for disadvantaged groups, to policy makers and to service providers.

## 1. Introduction

Traditional research dissemination through discipline-specific academic journals or conferences is increasingly under scrutiny as it reinforces passive relationships among research producers and users and fails to reach practitioners and policy makers who can translate findings into practice [1,2]. The approach hinders the adoption of innovative interventions, which increases the gap between research and practice.

Active dissemination is an alternative approach that involves a two-way dialogue between scientific researchers and non-academics to spread evidence-based interventions using planned strategies [3,4]. It is critical to the successful uptake of knowledge [5], may facilitate the successful incorporation of new evidence-based practices into routine care [6], and enable researchers to identify policy and practice priorities and relevant questions for future research [7], thus reducing the evidence-to-practice gap. The engagement of different types of stakeholders, including patients, practitioners and policy makers, is at the centre of active dissemination [8]. 

The community-based participatory research (CBPR) approach is a form of research that involves engaging with community stakeholders with the aim to improve the development, implementation and sustainability of evidence-based interventions [9,10]. Community-academic partnerships facilitate different voices and perspectives at all phases of the research process, from design to dissemination. The co-creation of knowledge via the engagement of relevant stakeholders in an active and committed decision-making process about a health issue is expected to lead to more meaningful research outcomes for patients, care providers, and communities [8,11,12]. 

The CBPR approach has been proposed as a promising framework to address health inequalities in socioeconomically disadvantaged (SED) communities [9,10]. In Ireland, a context of increasing lung cancer incidence in women [13] and higher smoking prevalence in SED groups compared to more affluent populations [14,15,16] led to the development of the We Can Quit (WCQ) intervention. WCQ is a peer-delivered smoking cessation intervention tailored to women from SED communities developed by the Irish Cancer Society (ICS), the Irish National Women Council and the Institute of Public Health in Ireland, following a CBPR approach [17]. It comprises group-based behavioural support, optional access to combination nicotine replacement therapy (NRT) without charge for all participants, and individual follow-up between group sessions [18]. 

The WCQ intervention was pilot tested in a community-based pilot randomised controlled trial (RCT), We Can Quit 2 (WCQ2) [18], which determined its acceptability and feasibility [19]. The engagement of non-academic stakeholders through establishing community-organised Local Advisory Groups (LAGs) was central to all phases of the WCQ2 pilot trial, from design to trial implementation. Trial stakeholders were patient/public representatives, community organisations, community pharmacists, primary care professionals, and practitioners from the ICS and the Irish Health Service Executive (HSE) [18]. 

The active dissemination of research that prioritises interactions with non-academic stakeholders is a core principle of CBPR [20]. However, literature reporting specific outputs from community dissemination is still scarce [8,11,20,21]. The contributions of non-academic stakeholders are described less frequently in reports on dissemination and implementation research [8]. Previous smoking cessation studies have used the CBPR approach to engage, implement, evaluate and disseminate interventions (see [22,23] for reviews). Only eight have evaluated interventions tailored exclusively to women, either during pregnancy [24,25,26,27] or to ethnic minorities [28,29,30,31], and just three were RCTs [24,26,30]. None of these studies describe how to ensure dissemination in communities or the specific outputs of this process [20,21,32]. The description of the dissemination activities and outcomes in a community setting in this study facilitates discovery of innovative ways to address common challenges, increases the visibility of contributions from community stakeholders, and generates insights on how to foster partnerships to develop culturally relevant research [2,20,21]. 

Knowledge exchange activities are the interactive interchange of knowledge between research users and research producers [33] used to disseminate scientific evidence [3]. A secondary objective of the WCQ2 trial was to develop strategies to optimise the recruitment and dissemination of findings to trial stakeholders to inform knowledge exchange and future research [18]. To achieve this objective, we reengaged with the WCQ2 pilot trial stakeholders as part of the trial dissemination plan and knowledge translation process. The dissemination plan included the design and distribution of an accessible policy brief summarising trial findings, and the process of conducting a knowledge exchange workshop. The workshop focused on capturing stakeholders’ views on trial findings and the experiences of being involved in research. It constituted a final feedback loop with stakeholders to outline priorities for future related research, the implications arising for policy and practice, and aimed to enhance the sustainability of the findings. The methods used and key lessons from this reengagement process are described in this paper.

## 2. Materials and Methods

### 2.1. Overview of the WCQ2 Pilot Trial

The WCQ2 pilot cluster RCT included an embedded mixed-methods process evaluation. The overall aim of WCQ2 was to determine the feasibility and acceptability of trial-related processes and of the WCQ intervention. A description of the trial protocol [18] and trial findings can be found elsewhere [19,34]. In brief, the pilot trial was conducted in four consecutive phases (waves), each one taking place in a randomly allocated matched pair of SED districts in Ireland [18]. LAGs were established in each district pair to support trial implementation, which primarily included on the ground establishment of the research sites and the planning and execution of the recruitment strategy. 

Each district was randomised to receive the WCQ intervention or a control treatment. WCQ comprised 12 weekly behavioural change group sessions delivered in a local community venue by trained lay, community facilitators from the local community who also provided optional access to combination NRT without charge for participants and follow-up one-to-one support on request. The control treatment comprised the HSE’s one-to-one smoking cessation service, with an average of 6/7 individual contacts delivered in person or by telephone by a smoking cessation officer. 

### 2.2. Design of an Accessible Policy Brief

An accessible policy brief (Supplementary Material File S1) was developed in collaboration with practitioners which included a plain English summary of the trial methodology and the principal findings and recommendations. It was widely distributed among local stakeholders involved in the conduct of WCQ2 and key regional and national practitioners and policy makers in Ireland with an interest in tobacco control. 

### 2.3. The WCQ2 Knowledge Exchange Workshop

#### 2.3.1. Pre-Workshop Planning, Design and Organisation

In collaboration with ICS and HSE practitioners, trial researchers designed a knowledge exchange workshop having completed data collection in September 2019. 

Workshop planning for two face-to-face workshops in May 2020 were postponed due to COVID-19 restrictions. The two face-to-face events were replaced by a single online workshop. The team compiled a list of invitees, who were contacted by email one month before the workshop. Invitees (*n* = 176) were those directly involved in the trial and those who had either expressed an interest in the research results or who had a remit for community healthcare and tobacco control. They included research partners, LAG members, representatives of the ICS and the HSE, programme delivery personnel (community facilitators and HSE smoking cessation officers), local area partnerships, community development organisations, community pharmacies, GPs, staff from primary care centres, regional and national policy makers and representatives of non-governmental organisations interested in tobacco control policies. All invitees received the accessible policy brief. Participation in the workshop was voluntary. 

Four PowerPoint presentations were developed to inform stakeholders of the main trial findings, including the lessons learned during recruitment from a community practitioner perspective and the experiences of delivering the HSE programme under trial conditions. Specific predefined topics were (1) the key improvements to enhance community engagement, participant recruitment and retention of women into the trial, which were lower than the target, to inform the design of a potential future definitive trial; and (2) the key policy and practice priorities arising from the WCQ2 research. Invitees could select to be assigned to one of two virtual breakout rooms in advance of the workshop.

#### 2.3.2. Workshop

The workshop was a two-hour online event delivered in November 2020. The PI, process evaluation trialist, and ICS and HSE practitioners delivered short presentations. 

Attendees were then assigned to one of the two breakout rooms to reflect on the predefined discussion topics. A researcher facilitated discussion (CBH and CD) in each virtual room using a structured question guide. They shared key insights at a final plenary session with all attendees. 

During the workshop, three researchers (SC, JV and CBH) collected field notes to obtain a descriptive overview of discussions on each workshop topic.

#### 2.3.3. Post-Workshop

The following day participants were invited by email to complete a separate online, anonymous, open-ended questionnaire (Supplementary Material File S2).

Researchers revised the field notes on the two days after the workshop. Thematic content analysis was used for field notes and questionnaire responses [35]. A researcher (SC) read all notes and manually coded passages to capture attendees’ views following predefined themes of interest: community engagement, recruitment, retention, and policy/practice priorities arising from the research. Subthemes for each of these categories were also identified.

## 3. Results

Forty-one stakeholders (41/176, 23% of total invitees) attended the event (Table 1), which represented an equal spread of community, regional and national stakeholders. Of these, 34 (83%) were directly involved in the set-up and delivery of the trial. The remainder represented cancer prevention, health promotion and improvement, local government, research and policy. Six attendees (6/41, 15%) completed the post-event questionnaire. Non-attendees (135/176, 76% of invitees) included research partners, community pharmacists, GPs, regional stakeholders and national policy makers with a role in tobacco use prevention and health promotion national programmes. Twelve of these (9%) sent apologies before the event, and six (4%) registered but did not attend. 

### 3.1. Themes Identified

#### 3.1.1. Community Engagement and Recruitment of Participants

The active mobilisation of LAG members to engage other community organisations and contact eligible women proved challenging [19]. Workshop attendees highlighted that the biggest challenge to community engagement was the limited time available to build strong trusted relationships with community partners and achieve broader engagement with the trial. Trial recruitment in four waves allowed an iterative learning process with adapted improvements implemented after each iteration. The plan for the final trial wave benefited from an extended nine-month engagement period with community stakeholders, a key factor in reaching the expected recruitment target [19]. 

Table 2 represents a summary of themes and subthemes arising from the analysis of field notes and questionnaire responses.

During the workshop, attendees highlighted the value of using various methods to promote the trial within each community. LAG members used direct and indirect strategies to reach eligible women from the general population in each target district: word of mouth, pop-up stands in community venues, leaflets, and social media advertisement [19]. The engagement of local stakeholders with in-depth knowledge of the community, including the community facilitators, enhanced recruitment rates. The availability of the principal investigator/research team member to discuss the programme in the communities and with primary care stakeholders was also recognised as essential to connect the fieldwork with research partners [19].

Workshop attendees also recognised the importance of engaging GPs and primary care staff at the earliest stages of the research process to enhance participant referral. In addition to the limited time available for engagement previously noted, attendees highlighted the challenge to maintaining participation of GPs and primary care workers during intervention delivery. The use of community social prescribing pathways to signpost participants to health and wellbeing programmes, such as the WCQ smoking cessation programme, was a potential solution recommended by attendees to support recruitment. Social prescribing is a pathway to link patients to nonmedical sources of support within a community, based on the recognition that social determinants of health have a significant influence on health outcomes [36,37]. It typically involves a link-worker (social prescriber) who acts as a link between the referrer (e.g., GP, nurse, other primary care professional) and the community resources.

#### 3.1.2. Retention

The typical reasons for participants’ dropout from the WCQ2 trial included feelings of not being ready or able to quit, or fear of admitting the need for help to quit smoking. Women’s families and social environment were also recognised as barriers to retention, through being regarded as a source of additional stress or by representing complex realities that may have hindered a woman’s participation. Lack of support and family members’ smoking behaviour may also have been a barrier. 

Workshop attendees recommended strategies to encourage attendance during intervention delivery. They suggested avoiding programme delivery during winter evenings or summer holidays as this was identified as a barrier to participation. They also recommended the inclusion of an ‘introduction night’ with former WCQ participants to share their experiences of programme delivery. They also suggested a dedicated week with participants’ families and friends during programme delivery to provide support and promote smoking cessation among women’s social networks. 

Workshop attendees also recommended keeping in contact with women by text or email one and three months after programme delivery to provide extra support and improve retention of participants at the longest follow-up point of the trial. They highlighted that some participating WCQ groups had organised walking groups to maintain social contact post intervention delivery. They recommended encouraging women to join other community programmes after programme delivery to maintain the group support and promote other healthy behaviours. 

In line with the pilot trial results [19], workshop attendees highlighted low literacy as a barrier to recruitment and retention of women into the trial. They recommended simplification of data collection forms and the participant information leaflet. Alternative methods to reduce the amount of reading materials, such as short videos, were also recommended. Attendees also suggested providing more support to complete data forms and more resources for community facilitators to address low literacy. 

#### 3.1.3. Policy Priorities Arising from the Research

Workshop attendees highlighted the need to remove cost and administrative barriers to access NRT to improve smoking cessation outcomes in SED populations and reduce smoking-related inequalities [38,39]. They also emphasised the need to prioritise funding for smoking cessation interventions tailored to lower socioeconomic groups, based in community settings. 

## 4. Discussion

The workshop successfully engaged a mix of community and statutory stakeholders representing different voices and perspectives who actively engaged in the proposed discussions. As a result, the trial team gleaned a set of practical strategies to enhance the design of future research implementation. Dedicated additional time to build relationships with local stakeholders with a tacit knowledge of their local context and connections to existing social prescribing networks, were recommended to enhance recruitment. Specific strategies to implement before, during and after intervention delivery, including measures to address low literacy levels, a week with family members during intervention delivery, keeping in regular contact with women and encouraging them to join healthy community programmes after intervention delivery, were suggested to encourage sustained participation and enhance the retention of participants at follow up. 

Sharing the findings with stakeholders and creating opportunity for feedback is crucial to contextualise data and constitutes a key step of the research process [1]. The dissemination workshop provided a final feedback loop with key trial stakeholders as a part of a longer community engagement process for the WCQ2 trial, which included ongoing interaction during the trial process with our PPI and community organisations. 

Workshops have an important role in facilitating knowledge exchange in CBPR research and may be an impactful tool to change policy and practice [5,22,40]. Previous community-based workshops or fora with stakeholders to disseminate health research findings have highlighted that workshops are resource-intensive activities as they require substantial funding, catering, supplies, and advertising [1,21]. Our dissemination strategy was planned to optimise the design of a definitive RCT, at the time of funding acquisition. The workshop was delivered online; hence no additional costs were incurred. Compared with other community-based events [2,21,41], it was of short duration (two hours). These characteristics may have facilitated attendance and may be easily replicated in other community-based studies and at different stages of a research project. 

Although we also targeted regional and national policy representatives with a wider brief in tobacco and cancer control, a majority of workshop attendees were community stakeholders, HSE and ICS representatives directly involved in trial planning and conduct (see Table 1). Hence, they reflected on trial findings based on their own experience of being part of the pilot trial, and on their in-depth understanding of the broader community context. Joint reflection among non-academic stakeholders and researchers facilitated new insights and a deeper understanding of what is required to address the needs of disadvantaged women to allow them to fully participate in a smoking cessation trial and smoking cessation services in their local communities. Their participation ensured a community-based input to co-create action strategies to address the challenges found during trial implementation in community engagement, recruitment and retention. 

The importance of community engagement to trial outcomes has been referred to by other community-based RCTs recruiting marginalised groups of women [30,42]. Our paper highlights the time involved in developing trusted relationships with community stakeholders, a common challenge in the conduct of CBPR [21,42]. Building relationships with community stakeholders requires the dedicated support and resources needed to successfully identify community champions, and to develop strong partnerships within a community, all of which must be considered in the design stage of a trial [30,43]. 

The recruitment of participants in SED populations to health research studies is challenging. It is dependent on the active engagement of local stakeholders [19]. A systematic review of the barriers to sampling, recruitment and retention of SED groups in health research suggested that health professionals fail in participant referral as they do not have the time to recruit or may have poor communication skills [44,45]. In addition to the broader engagement of primary care professionals for participants’ referral, connecting with established social prescribing networks in community settings may be an important strategy to enhance recruitment rates. This would also support retention at follow-up by strengthening the links between participants and other available community resources. 

The challenge of retaining participants from SED populations in smoking cessation trials has been recognised as being influenced by individual and contextual factors [38,44], and dependent on the use of multiple techniques, including contact tracing, data collection in community locations, and incentives and gifts [46]. In addition to addressing participants’ low literacy, lack of support and the smoking behaviour of their social networks identified from the workshop, these additional strategies may be useful to improve retention in future related research. Keeping in contact with participants after intervention delivery may help to build rapport and promote trust and commitment to the trial [45], and translate into higher data completion rates at follow-up [44]. 

The inclusion of these recommended individual, community and population level strategies to enhance community engagement, recruitment and retention may hold the key to improve smoking cessation rates among SED smokers [46,47,48,49]. The value of these strategies may extend beyond a research setting and enhance sustainability of real-world smoking cessation services. The existence of the LAG infrastructure may assist in the implementation of other community-based health interventions. The knowledge gained from the conduct and dissemination of this research may provide useful learning for those involved in policy development addressing health inequalities. 

The current dissemination plan has important limitations. The workshop took place a year beyond completing data collection, which may have negatively impacted stakeholders’ participation and recall. In retrospect, arranging a second online workshop may have attracted higher attendance and wider stakeholder participation from those not involved in the trial conduct. This would have facilitated an informed view of the barriers and facilitators to applying a similar model for smoking cessation in other contexts. 

At the start of the pandemic when the workshop took place, live recording of online discussions was not commonplace, and issues of consent were not fully worked through. Hence, workshop discussions were not audio recorded. While reliance on field notes may have in theory have hindered the comprehensive registration of data, we believe that our practice of using multiple researchers to combine notes in a timely manner after the event to facilitate recall, sufficiently mitigated against this. 

The method applied in our study as well as the low response rate to the post-workshop questionnaire (15%) may have led to a serious risk of bias [50]. We did not include any formal measures to determine the impact of our dissemination activities on policy and practice. Nevertheless, the HSE stated their intention to follow up promising patient outcomes and recommendations on practical implementation, as WCQ has now been included as a programme under the national Healthy Ireland Strategic Action Plan 2021–2025 [51] to address health inequalities, by focusing on promoting healthy actions amongst disadvantaged and harder to reach communities. The development of accessible recommendations tailored to the target audience, and our recognition of the critical role and value of engaging stakeholders in all phases of the research, may have helped to overcome poor communication and any feelings of mistrust [7]. These results demonstrate the importance of co-creating and conducting community-engaged research that is locally relevant. These elements are likely to improve impact of the research findings that may be crucial to address smoking cessation in lower SED women [20].

## 5. Conclusions

The dissemination of results following the CBPR approach encouraged the joint reflection of community, statutory and academic stakeholders on new research findings. It increased the understanding of barriers and facilitators to SED women’s engagement in a smoking cessation intervention, and assisted in developing recommendations and outlining policy priorities that can be implemented in practice. We expect our findings to be generalisable to the development of other community-based behavioural change interventions in lower SED groups. The findings may be used to optimise the design of definitive RCTs to test the effectiveness of these interventions, and to enhance their sustainability, thus contributing to a reduction in health inequalities. Despite the low response rate, the online approach was a successful medium for stakeholder engagement and may be useful approach for future knowledge exchange and dissemination of community based research. 

## Figures and Tables

**Table 1:** List of workshop participants including their professional role and involvement in trial conduct.

Attendees (*n* = 41)	Involved in Trial Planning and/or Delivery	Description
HSE and ICS representatives (*n* = 7)	Yes	Delivery partners involved in setting up Local Advisory Groups and delivery of the recruitment in each trial district. HSE representatives who were experts in smoking cessation and trained the trial programme delivery personnel.
Programme delivery personnel (*n* = 9)	Yes	Community facilitators and HSE Smoking cessation officers who delivered trial interventions. They were also involved in retention during intervention delivery.
Local Advisory Group members (*n* = 18)	Yes	Representatives from Community development organisations, Local Area Partnerships, and Primary Care workers who were involved in trial planning and in the promotion and delivery of the recruitment strategy. Local Authority representatives from the cities and counties in which the trial took place, which were involved in planning trial delivery.
Regional Policy Makers (*n* = 3)	No	HSE Community Health Officers, Primary Care Development and Health Promotion representatives from trial areas.
National Policy Makers (*n* = 4)	No	Representatives from the tobacco and cancer prevention national programmes in Ireland. Representative from a voluntary organisation working to reduce tobacco use and related disease. Representative from a public education charity interested in addressing inequalities.

**Table 2:** Themes and subthemes from field notes and questionnaire responses.

Predetermined Theme	Subthemes
Community engagement and participant recruitment	Increase the research set-up time to build trust relationships with community stakeholders. Variety of methods to recruit participants. Increase the engagement of GPs and primary care workers. Participants’ low literacy levels to interpret research information.
Retention	Reasons for discontinuing with the research. Tools for encouraging attendance during the programme delivery. Planned support after intervention delivery to reinforce quit attempts and improve the collection of data at 6 months. Participants’ low-literacy-programme materials.
Policy priorities arising from the research	Barriers to access NRT. Smoking cessation in disadvantaged groups.

## Data Availability

The data are not publicly available to preserve the identity of participants.

## Supplementary Materials

The following supporting information can be downloaded at https://www.mdpi.com/article/10.3390/ijerph19042333/s1, Supplementary Material File S1: WCQ2 policy brief; Supplementary Material File S2: Questionnaire post-event.
