# Supplementary material for: The We Can Quit2 Smoking Cessation Trial: Knowledge Exchange and Dissemination Following a Community-Based Participatory Research Approach
Source: Int J Environ Res Public Health. 2022 Feb 18;19(4):2333. doi: 10.3390/ijerph19042333 (PMC8872427; doi:10.3390/ijerph19042333)
Supplement: Supplementary file 1 [file ijerph-19-02333-s001.zip › ijerph-1494282-File S1.pdf]

File S1 – WCQ2 policy brief

Policy Brief

We Can Quit2: Results of a pilot cluster randomized controlled trial of a community-based intervention on smoking cessation for women living in disadvantaged areas of Ireland

Castello S,[1] Darker C,[1] Vance J,[2] Buckley E,[3] Reynolds C,[3] Buggy A,[3] O'Hagan K,[2] Cronin N,[4] Dougall N,[5] Devane D,[6] Bauld, L,[7] Hayes CB[1].
[1]Public Health & Primary Care, Institute of Population Health, Trinity College Dublin, Ireland [2]Irish Cancer Society, Ireland [3]Health Service Executive, Ireland [4]National Women's Council, Ireland [5]School of Health and Social Care, Edinburgh Napier University, UK [6]HRB Trials Methodology Research Network & NUI Galway, Ireland [7]Usher Institute, University of Edinburg, UK.

Health consequences of smoking tobacco

Tobacco use is the leading cause of preventable death worldwide and is a causal factor for many chronic diseases and types of cancer. Ireland ranks second highest for smoking-related causes of death in the EU.

Strongly linked to smoking, lung cancer is now the most common cause of death for women in Ireland, having surpassed breast cancer rates. These health consequences of smoking tobacco impact more in low socio-economic status (SES) groups. This reflects the need to find more effective ways to engage women smokers in smoking cessation services.

In response to this, the Irish Cancer Society (ICS) in collaboration with the National Women's Council Ireland, the Institute of Public Health and the Health Service Executive (HSE) in Ireland developed We Can Quit (WCQ). It is a community-based stop smoking programme specifically designed for women living in socio-economic disadvantaged areas in Ireland.

Key components of the WCQ programme

- ⇒ Group-based support once a week for 12 weeks, delivered by Community Facilitators (CFs): lay trained women living or working in target areas.
- ⇒ Access to Nicotine Replacement Therapy (NRT) without charge for participants who wish to take it.
- ⇒ Individual one-to-one text support between sessions.

Our Study: the We Can Quit2 pilot trial (study)

The **We Can Quit2** trial **tested if it was feasible (possible) to recruit eight community districts (four matched pairs) to a stop smoking trial**; if we could recruit **194 women in total (48-49 women in each matched pair of districts)**; if we could **randomly assign** one district in each matched pair (24-25 women) to the WCQ programme and one to the face to face smoking cessation services (usual care) provided by the HSE when recruitment was complete; and if we had **2/3 of the women who were recruited to the study remaining at the end of the programme**. We examined **if the process of being involved in a trial was acceptable to the women and the CFs and what they thought of the WCQ programme itself**. Based on our findings, **we recommend that a full effectiveness trial** comparing quit rates in the two groups is needed. **This future trial would allow us to inform and improve future smoking cessation services delivery targeted to low-income groups.**

What did we do?

- ⇒ We recruited districts (four clusters) defined as socio-economically disadvantaged according to the National Deprivation Index (Pobal), which were geographically proximal and with available one-to-one smoking cessation services provided by the HSE.
- ⇒ We set up Local Advisory Groups (LAGs) involving local area Partnerships, the HSE, ICS representatives, community development organisations, pharmacies and GPs. They actively engaged local stakeholders to recruit women, and assisted in planning the trial in each pair of districts.
- ⇒ We tested the recruitment plan, using an iterative process over four waves.
- ⇒ Members of the LAGs contacted eligible women through social media, word-of-mouth, community stands, and leaflets/posters.
- ⇒ We applied inclusion criteria which included a target of 50% medical cardholders. We allocated (randomised) women into one of two trial arms: intervention (the WCQ programme) or control (usual one-to-one HSE smoking cessation service).
- ⇒ At the end of the 12-week intervention and at 6 months, we administered questionnaires to collect the following data from women in both arms: eligibility and recruitment rates; engagement with smoking cessation services and attendance rates; data completion and retention rates. Acceptability of the trial processes and of the WCQ programme was determined mainly at interview with participants and CFs. We also obtained data on smoking abstinence and physical and mental health.

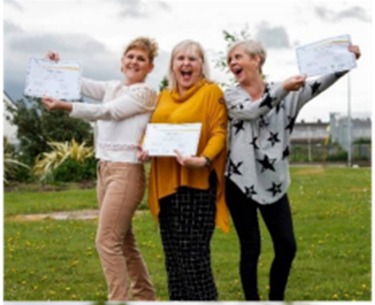

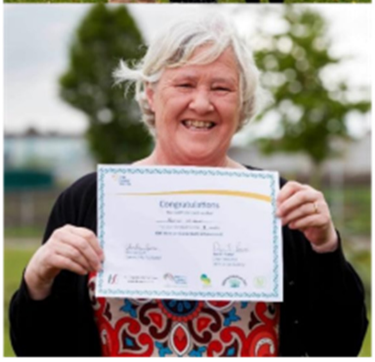

This trial was funded by the Health Research Board Ireland under the Definitive Interventions and feasibility Awards DIFA-2017-048.

Trinity College Dublin Coláiste na Tríonóide, Baile Átha Cliath The University of Dublin | Irish Cancer Society | Edinburgh Napier UNIVERSITY | TMRN | HRB Health Research Board

Our findings

Successful recruitment of districts and participants

We successfully recruited four pairs of districts and 125 of the 208 eligible women consented to participate. In the final wave we achieved the recruitment target of 49 women.

Characteristics of women in the study

Socio-demographic: The average age was 48 years old. Almost half were not in a paid employment and had only primary/no formal education. Two-thirds were medical cardholders.

Smoking habits: Women smoked on average 18 cigarettes a day. Habit or addiction were the most frequent reasons given for smoking. They were very determined to quit. Most intervention women smoked for over 25 years.

Engagement, attendance and retention

- Engagement (i.e. having attended at least one session and set a quit date) was a 12% higher in intervention women.
- Women who quit smoking in each group attended over twice as many sessions than those women who continued smoking.
- Approximately half of recruited women provided follow-up data at 12 weeks and/or 6 months.

Trial processes and the WCQ programme are acceptable

- Interview data indicated that trial processes including randomisation and data collection were acceptable. Although all forms were literacy proofed, literacy was cited as a problem by a small number of women.
- Most women in the WCQ group used NRT, and patches were the most used NRT type. Removing cost as a barrier for using NRT was welcome. Women recalled positive reinforcement by other women and learning from each other were highly helpful in motivating them. They highlighted the support given by the community pharmacist for their quit attempt.
- The CFs delivered the session content as planned. A few remarked the big volume of paperwork to fill after each session under trial conditions.

"I don't write very well. So, what we were doing was em [XX] would help us with the filling out so you don't feel embarrassed… it is embarrassing when they're asking us to fill in stuff which I can't do..." (P13)

"… it's encouragement and listening to their stories and to be able to say I can take that on board, and you end up saying 'I can do that as well'" (P03)

"…given it was the research programme and everything, there was lots of extra stuff to do…" (CF02)

"It was great, yeah, yeah, I found it fantastic" [NRT without cost] (P49)

Other findings

Although not set up to test this in a small pilot trial, we observed that more WCQ women had stopped smoking at the end of the programme and smoked fewer cigarettes per day than women attending usual care. WCQ women also reported having better physical health at 12 weeks and improved mental health at 6 months. These findings need to be tested in a larger trial before we can be certain that they are true.

Conclusions

- We successfully reached low SES women to the study through the work of the LAGs and community mobilisation, so recruitment of women to a community based smoking cessation trial was feasible but challenging.
- Women engaged well with their assigned programme. Removing the cost barrier to NRT access was a success factor of the WCQ intervention. Keeping women in the study (retention) benefited them, however retention rates were lower than expected. These issues and literacy issues need to be addressed in the future trial designed to test effectiveness.

Recommendations for policy and future research

Ensuring successful recruitment in a future trial

- Dedicate more planning time for intervention set-up and development in each district.
- Engage a representative of the Community Health Organisation Primary Care Team/Network in the LAGs.
- Resource a local coordinator to support recruitment in communities.
- Work on a referral system to make easier for GPs and healthcare workers to register potential participants.

Improving participants' retention

- Implement an intervention boost a month after programme end, to maintain contact with and support participants during and after the programme.

Addressing literacy issues

- Offer assistance to complete all forms from the first contact with any potential participant.
- Include videos to explain consent and all contents/measures.
- Increase training for CFs on strategies to support women dealing with these difficulties.

Removing barriers to NRT use/access

- Make NRT universally available free of charge to participants in all HSE recognised smoking cessation programmes. Remove administrative barriers to access NRT treatment for current medical cardholders. This would encourage participation and lead to higher smoking abstinence rates in disadvantaged areas.

Further information: Hayes C, Ciblis A, Darker C, Dougall N, Vance J, O'Connell N, Dobbie F, Loudon K, Burke E, Devane D, and Bauld L. We Can Quit2 (WCQ2): a community-based intervention on smoking cessation for women living in disadvantaged areas of Ireland—study protocol for a pilot cluster randomised controlled trial. Pilot Feasibility Stud 5, 138 (2019). https://doi.org/10.1186/s40814-019-0511-9
