# Supplementary material for: The We Can Quit2 Smoking Cessation Trial: Knowledge Exchange and Dissemination Following a Community-Based Participatory Research Approach
Source: Int J Environ Res Public Health. 2022 Feb 18;19(4):2333. doi: 10.3390/ijerph19042333 (PMC8872427; doi:10.3390/ijerph19042333)
Supplement: Supplementary file 1 [file ijerph-19-02333-s001.zip › ijerph-1494282-File S2.pdf]

File S2. Questionnaire post event

Key Improvements to enhance community engagement and participant recruitment and retention in future research trials.

A. Community engagement.

1. In your experience/from your perspective, which were the key challenges and opportunities in getting community members engagement over time in the We Can Quit2 study?

B. Recruitment of participants.

2. Which were the biggest challenges to recruitment of participants?
3. How might participants recruitment be improved in a future study?

C. Retention: keeping women in the study.

4. Why do you think women dropped out?
5. What suggestions do you have to improve participants retention?
6. Are there any practices at an organisational level that may improve retention?

Key policy and practice priorities arising from the We Can Quit2 study.

A. We Can Quit2 study findings and policy.

1. What do you think are the most relevant findings of the We Can Quit2 study from a policy and practice perspective?
2. What do you think on the Policy Brief recommendations?

B. Recommendations for policy makers arising from the study findings.

3. What challenges or barriers might arise in attempting to achieve recommendations on the policy brief?
4. What kind of support would be needed in working with communities to achieve these recommendations?

C. Disseminating the findings at a policy level.

5. Thinking on people, organisations, community groups you may know. Who needs to know about the study?
